# Pro-apoptotic and anti-adhesive effects of four African plant extracts on the breast cancer cell line MCF-7

**DOI:** 10.1186/1472-6882-14-334

**Published:** 2014-09-09

**Authors:** Nadja Engel, Abiodun Falodun, Juliane Kühn, Udo Kragl, Peter Langer, Barbara Nebe

**Affiliations:** Department of Cell Biology, University Medical Center Rostock, Schillingallee 69, 18057 Rostock, Germany; Department of Pharmaceutical Chemistry, Faculty of Pharmacy, University of Benin, Benin City, 300001 Nigeria; Institute of Chemistry, University of Rostock, Albert-Einstein-Str. 3A, 18059 Rostock, Germany

**Keywords:** Traditional medicine, Breast cancer, Plant extraction, Cell cycle, Apoptosis, Integrin, Adhesion, Anoikis

## Abstract

**Background:**

*Jatropha curcas* (JCP1), *Pyrenacantha staudtii* (PS), *Picralima nitida* (ZI) and *Jatropha gossypifolia* (JCP2) are plants used in the African folklore for the treatment of various cancers.

**Methods:**

This study investigated the *in vitro* anticancer effects of the ethanol extracts against human epithelial MCF-7 breast cancer cells in a dose-dependent manner (1–50 μg/ml) by using cell cycle analysis, viability assay, annexin V/PI staining, TUNEL method and expression determination of apoptotic and adhesion relevant proteins. Adhesion processes were monitored by detachment via flow cytometry, β1-integrin expression and formation of the actin cytoskeleton.

**Results:**

The three extracts, termed PS, JCP1 and JCP2 at a concentration of 10 μg/ml induced cell death in MCF-7 breast cancer cells verified by high amounts of PI-positive cells in the cell cycle analysis, Annexin V/PI staining and DNA fragmentation measurements. In parallel cell detachment was accompanied by decreased β1- integrin expression and phosphorylation of the focal adhesion kinase at Tyr397. ZI extract was the exception by the increasing β1-integrin expression and strengthening the cortical actin cytoskeleton. However, all four plant extracts mediated strong anti-cancer properties with IC_50_ values between 23–38 μg/ml.

**Conclusion:**

PS, JCP1 and JCP2 were found to be very active against MCF-7 cells by inducing anoikis and therefore possessing vast potential as medicinal drugs especially in estrogen receptor positive breast cancer treatment. ZI mediated their anti-cancer action by different signaling mechanisms which should be analyzed in future studies. Our results further supported the idea that medicinal plants can be promising sources of putative anticancer agents.

**Electronic supplementary material:**

The online version of this article (doi:10.1186/1472-6882-14-334) contains supplementary material, which is available to authorized users.

## Background

The use of natural products including medicinal plants has become more and more important in primary health care especially in developing countries. Many pharmacognostical and pharmacological investigations are carried out to identify new drugs or to find new lead structures to develop novel therapeutic agents for the treatment of human diseases such as cancer [1]. In developing countries and particularly in Yemen, a large segment of the population still rely on folk medicine to treat serious diseases including infections, cancer and different types of inflammations.

Currently, there is insufficient scientific research on the plants from Nigeria. Previous studies described the anticancer investigations of some endemic and non-endemic plants from Nigeria [2]. This study was carried out as a part of our continued exploration of Nigerian medicinal plants for interesting biological activities. Thus, the main aim of the present project was to carry out a phytochemical and cell biological investigation on selected plants from the southern part of Nigeria, especially on those that are endemic and those that find a use in traditional medicine as anticancer agents. In this study, four plants were collected for evaluation of their antitumor activities with respect to pro-apoptotic and anti-adhesive properties.

*Picralima nitida* (Stapf.) Th. & H. Durand (Flowering plant family: *Apocynaceae*) has widely varied applications in Nigeria folk medicine for antipyretic, antihypertensive, gastro-intestinal disorders, as an antimalarial, aphrodisiac, antitrypanocidal, and as a remedy against hyperglycemia [3–6]. The plant is used in Nigeria and West Africa as remedy against breast cancer [7].

*Pyrenacantha staudtii* Hutch and Dalz (Tropical forest tree family*: Icacinaceae*) is a medicinal plant widely used in tropical Africa for the treatment of various ailments such as stomach disorders, intestinal colic, menstrual disorders, and as anticancer and antiabortificient agents [8, 9].

*Jatropha gossypifolia* L (*Euphorbiaceae*) is widely distributed in many tropical countries [10, 11]. It has antibacterial, antiinflammatory, analgesic and anticancer activities [12]. It was known in ancient medicine for its ethnomedicinal uses in the treatment of cancerous growth and for its pesticidal activity [13, 14].

*Jatropha curcas* Linnaeus (*Euphorbiaceae*) is a small tree or large shrub that can reach a height up to 5 m. It is used in traditional medicine as remedy against cough, cancer, and human immunodeficiency virus [15]. The local populace in the eastern part of Nigeria uses the ethanol extract for the treatment of breast cancer. In some cases, traditional herbal practitioners use the aqueous decoction to cure cancer [15]. The search for anticancer agents via activity directed identification and characterization lead to the present study with a view to validating the claimed ethno-medicinal property of these plants as anticancer remedy. Hence, this study is focused totally on the exposure of the ethanol root bark extracts of the plants to MCF-7 cancer cell line in a dose-dependent manner.

## Methods

### Collection and identification of plant materials

The plant parts (listed in Table 1) were collected from different locations of Nigeria in the rainy season (March-June) of 2011 and identified by Mr. A. Sunny of the Department of Pharmacognosy, Faculty of Pharmacy, University of Benin, Benin City. Voucher specimens are deposited at the Faculty of Pharmacy, University of Benin, Nigeria.

**Table 1:** **Properties of the four Nigerian plants used in this study**

Plant	Abbr.	Family	Parts used	Medical uses	Location	IC _50__MCF-7_ (μg/ml)
*Jatropha curcas Linn	JCP1	Euphorbiaceae	RB	cough, wound healing, HIV, cancer	Benin City	36.55
*Pyrenacantha staudtii Hutch & Dalz	PS	Icacinacaeae	L	threatened abortion, malaria, GIT and cancer	Benin City	37.36
*Picralima nitida Th. & H. Durand	ZI	Apocynaceae	RB	malaria, hyperglyaceamia, antiseptic etc.	NIFOR	22.76
*Jatropha gossypifolia Linn	JCP2	Euphorbiaceae	RB	cancer, pesticides	Owan	25.55

Overview of the four plant extracts including its medical uses, IC_50_ values at 48 h for MCF-7 cells. RB; Root bark; L; leaf; * Most of the information of traditional use has been taken from native people.

### Preparation of plant extracts

The powdered plant samples (100 g) were each extracted by maceration, with ethanol (250 ml) at room temperature, and concentrated to dryness using a rotary evaporator at reduced pressure. The% yield (10, 23, 40 and 51 for JCP1, PS, ZI and JCP2, respectively) was obtained. Dried samples were stored at −20°C until further use. Finally, all plant extracts were dissolved in dimethylsulfoxide (DMSO) to give a desired stock solution of 50 mg/ml, which was aliquoted and stored at −80°C.

### Phytochemical composition of extracts

The ethanol extracts were subjected to photochemical screening in order to identify the secondary metabolites and nature of the extracts. The method employed, was from Trease and Evans [16].

### Cell culture

Human breast adenocarcinoma cell line MCF-7 (ATCC no. HTB-22) was obtained from the America Type Culture Collection (Manassas VA, USA). Cells were maintained at 37°C and in a 5% CO_2_ atmosphere in a monolayer in Dulbecco’s modified Eagle’s medium (DMEM, Invitrogen, Germany) with 10% fetal bovine serum (PAA Laboratories GmbH, Germany) and 1% gentamycin (Ratiopharm, Germany). Confluent cells were passaged by treating them with 0.05% trypsin/ 0.02% EDTA. The medium was changed every two days. MCF-7 cells were authenticated by morphology and growth rate and were mycoplasma free. Cultivation conditions were described previously [17].

### Treatment with plant extracts

Treatment conditions were previously described [17]. Treatments with the four plant extracts (final concentrations of 1, 10, 25, 50 μg/ml) were carried out for 48 h in assay medium. As negative control substance the vehicle dimethylsulfoxide (DMSO, 0.1%) was used in the same manner.

### Cell cycle analysis

To determine proliferation and apoptosis alterations, the cell cycle analysis via flow cytometry (FACSCalibur, BD Biosciences) after propidium iodide staining (50 mg/ml) of the MCF-7 cells was carried out [17, 18]. For data acquisition, the software FlowJo version 7.6.5 (Tree Star; http://www.flowjo.com) was used. A minimum of 15,000 ungated events were recorded. For statistical analysis, the S-phase and G2/M-phase cells of the cell cycle were defined as proliferative cells and the sub-G1-peak of the histogram as apoptotic ones.

### Annexin V/PI apoptosis detection

In this assay, Annexin-V detects the translocation of phosphatidylserine from the inner leaflets to the outer leaflets of the plasma membrane, which is a key feature of apoptotic cells, whereas PI detects necrotic cells with permeabilized plasma membrane. Labeling of early apoptotic and dead cells was performed according to the manufacturer's instructions from the Alexa Fluor488 Annexin V/Dead Cell Apoptosis Kit (Thermo Fisher Seintific Inc., Germany). Cells were treated with 10 μg/ml plant extract for 48 h. After treatment detached as well as adherent cells were washed twice with cold PBS. The cell pellet was resuspended in 100 μl of annexin binding buffer at a density of 1 × 10^6^ cells per ml and incubated with 5 μl of Alexa488-conjugated Annexin-V and 5 μl of PI for 15 min at room temperature in the dark. 400 μl of 1× binding buffer was added to each sample tube, and the samples were immediately analyzed by flow cytometry. Histograms and statistics were designed with the software FlowJo Version 7.6.5.

### Calculation of cell detachment

400,000 MCF-7 cells were seeded in 6-well plates (Greiner, Germany). After treatment with the four plant extracts (10 μg/ml) and the DMSO control detached cells were counted by flow cytometry.

### Measurement of integrin expression

Measurement and calculation of β1-integrin expression at the cell surface by flow cytometry (FACSCalibur) was described previously [18]. Anti-integrin antibody β1 (CD29; Immunotech, 0.2 mg/ml, mouse anti-human 4B4, Isotype: IgG1) was secondarily labeled with fluorescein isothiocyanate-conjugated anti-mouse IgG (Fab_2_) fragment (Sigma). Ten thousand events were recorded for each measurement and each measurement was repeated three times.

### Immunofluorescence and microscopy

Filamentous (F)-actin was selectively labeled with BODIPY® FL phallacidin emitting green fluorescence (Invitrogen, Germany). Nuclei were stained with Hoechst dye (Invitrogen, Germany). Bright field and all fluorescence images were obtained using Axio Scope A1 fluorescence microscope (Carl Zeiss, Germany). Individual fluorophores were imaged in black and white for maximum sensitivity and pseudocolored and overlaid using AxioVision Imaging Software Release 4.8.2. (Carl Zeiss, Germany).

### Western blotting procedure

After treatment with the plant extracts for at least 48 h the cells were trypsinized, washed with PBS and lysed in ice-cold lysis buffer (Bio-Plex Cell Lysis Kit, Bio-Rad, USA). Cells were homogenized by brief sonification at 4°C and centrifuged at 10,000 g for 2 min at 4°C. Protein concentrations of the supernatants were estimated by Bradford protein assay so that equal amounts (10 μg) of total soluble protein could be separated by Criterion TGX Stain-Free precast gels (Bio-Rad, Germany) and blotted on PVDF membranes. After SDS-PAGE, protein content per lane as well separation quality was additionally controlled with the Criterion Stain FreeTM gel imaging system (Bio-Rad, Germany). After the protein transfer membranes were blocked with 5% skim milk in Tris buffered saline (TBS) and washed six times in TBS. For protein detection primary antibodies (β1 integrin: sc- 374429; PCNA: sc- sc-56; both from Santa Cruz, USA; caspase 7, 8, 9 from the Apoptosis sampler kit #9915; FAK antibodies within the sampler kit #9330; Akt #4691; pAkt (S473) #9271; p44/42 MAPK #9102; P-p44/42 MAPK (T202/204) #4377; β-Actin #4970: all from Cell Signaling, USA) were incubated overnight at 4°C followed by labeling with a horseradish peroxidase (HPR)-conjugated secondary antibody (Dako, Glostrup, Denmark) for 1 hour at room temperature. Protein signals were visualized by using SuperSignal West Femto Chemiluminescent Substrate (Pierce Biotechnology, Rockford, USA) for detection of peroxidase activity from HRP-conjugated antibodies (Thermo Fisher Scientific Inc., Rockford, USA). Band intensity was analyzed densitometrically with the Molecular Imager ChemiDoc XRS and Image Lab 3.0.1 software (Bio- Rad, USA). Protein detection was repeated at least three times with individual prepared cell lysates from independent passaged cells.

### Tunel assay

Apoptotic DNA degradation was stained using the terminal deoxynucleotidyl transferase (TdT)-mediated dUDP-biotin nick end labeling (TUNEL) method. In this study, the In Situ Cell Death Detection Kit, Fluorescein (Roche, USA) was used for this purpose according to the manufacturer’s protocol. Briefly, MCF-7 cells (1.5 × 10^6^ cells/well) were cultured on cover glasses in 6-well plates. After exposure to the plant extracts, cells were washed with PBS, fixed with 4% paraformaldehyde solution for 1 h at 15–25°C, and incubated in permeabilisation solution for 2 min on ice. After washing with PBS cells were incubated with the TUNEL reaction mixture for 60 min at 37°C in a humidified atmosphere in the dark.

Fluorescence of the stained cells was observed using a Carl-Zeiss confocal laser scanning microscope (LSM 780, Jena, Germany) with an excitation wavelength of 488 nm.

### Calculation of IC_50_ values

The half maximal inhibitory concentrations (IC_50_) values were calculated by colorimetric measurements of mitochondrial metabolic activity with the CellTiter MTS/PES assay following to the manufacturer’s instructions (Promega Corp., Madison, WI). MTS is a tetrazolium compound [3-(4,5-dimethylthiazol-2-yl)-5-(3-carboxymethoxyphenyl)-2-(4-sulfophenyl)-2H-tetrazolium] which is combined with an electron coupling reagent (phenazine ethosulfate; PES) to form a stable solution. The conversion to formazan is bioreduced by cells which is presumably accomplished by NADPH or NADH produced by dehydrogenase enzymes in metabolically active cells. The measured mitochondrial metabolic activity also reflects the cell cytotoxicity directly and the cell viability indirectly.

Cells were seeded in 96-well plates at a density of 2000 cells/well in 100 μl medium and left to attach for 24 h. Treatment with plant extracts at final concentrations of 1, 10, 25, and 50 μg/ml was carried out as described previously. In parallel, control approaches were carried out with medium only and 0.1% of the solvent DMSO to calculate background absorbance. No background absorbance was obtained for the extracts and MTS in the absence of cells, as some extracts are capable of reducing the MTS. After an initial incubation for 24 h cells were assayed with MTS according to the manufacturer’s instructions (Promega Corp., Madison, WI). Colorimetric changes were measured at 490 nm and raw data was transferred to Microsoft Excel and analyzed. At least 8 replicates corrected with the background absorbance were performed. Reduction of cell viability at each concentration was plotted as a dose response curve. The IC_50_ of the active extracts were calculated using nonlinear regression to fit data to the dose–response.

### Live/dead cell staining

Live/Dead cell staining was performed following the manufacturer's instructions (Live/Dead Cell Staining Kit II, PromoCell GmbH, Germany).

### Statistical analysis

All data were analyzed by the Students *t*-test using Microsoft Excel 2010. Every experiment was done in triplicate with individual passaged cells and data sets were expressed as means ± standard deviations (SD). Statistical significance was represented as ***P < 0.001, **P < 0.01, *P < 0.05.

## Results and discussion

The list of the investigated plants, the parts used and their known medicinal uses are represented in Table 1. These informations were sorted out in accordance with the recommendation of the *Handbook of African Medicinal Plants* [4]. *Ethnobotany Desk Reference* [19], and direct information obtained by Dr. A. Falodun (Department of Pharmaceutical Chemistry, Faculty of Pharmacy, University of Benin, Benin City, Nigeria) through interviewing local traditional healers. Ethno pharmacological data (information based on the medicinal traditional use of plants) has been one of the common useful ways for the discovery of biologically active compounds from plants [20]. The phytochemical composition of the extracts showed the presence of alkaloids, tannins, saponins, flavonoids in JCP1, PS and ZI. Only tannins and flavonoids were present in JCP2 (Table 2).

**Table 2:** **Determined substance classes**

Phytochemical compositions				
	***J. curcas***(JCP1)	***P. staudtii***(PS)	***P. nitida***(ZI)	***J.gossypifolia***(JCP2)
Alkaloids	+	+	+	-
Saponins	+	+	+	-
Tannins	+	+	+	+
Flavonoids	+	+	+	+

Phytochemical compositions of the root bark extracts of four medicinal plants. + presence of components; − absence of components.

### Proliferation and apoptosis events after treatment with the plant extracts

**Figure 1:**
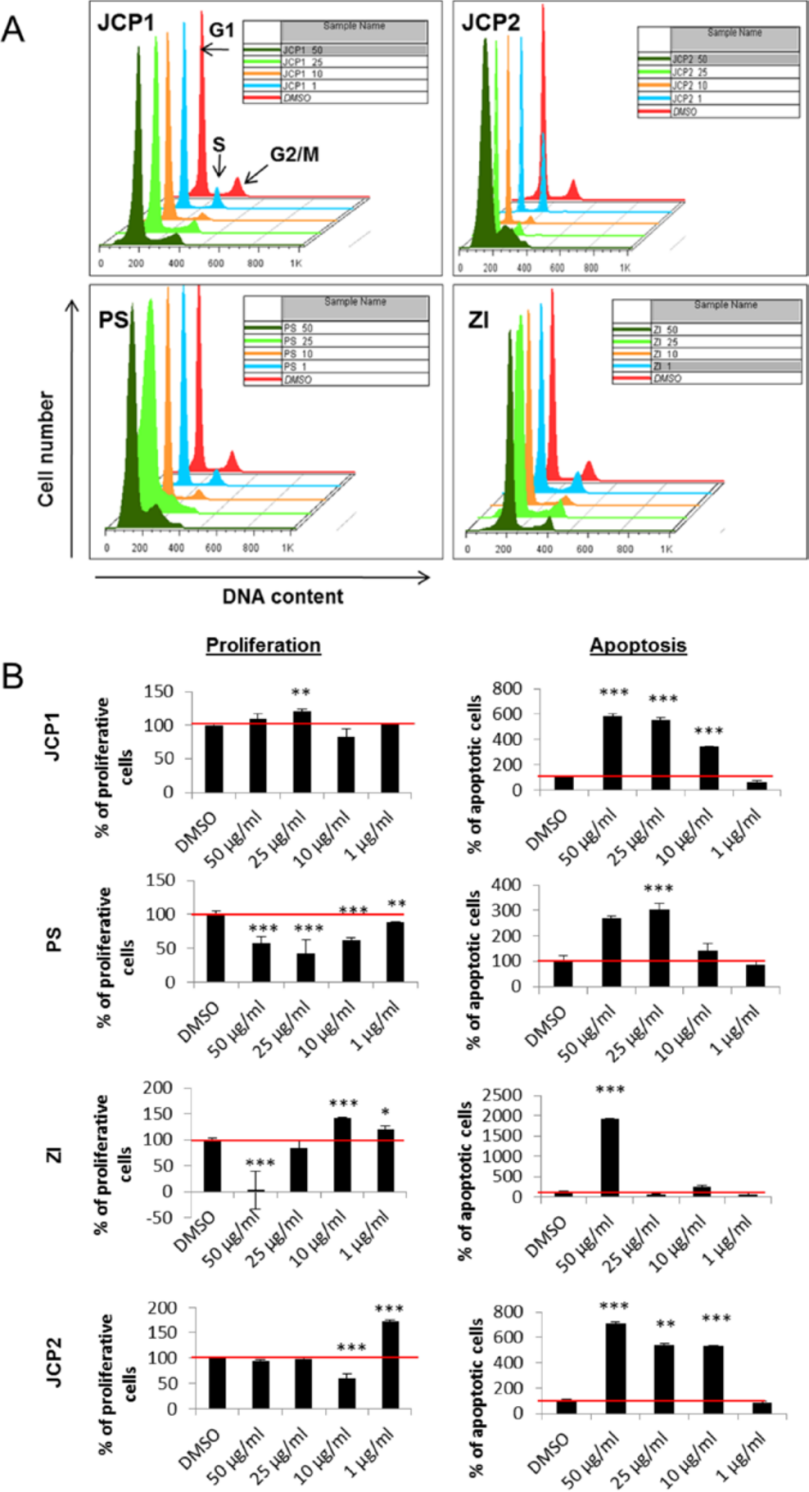
**Cell cycle analysis of MCF-7 cells. A**: Histograms of the cell cycle distribution of MCF-7 cells after treatment with the control substance DMSO and the plant extracts JCP1, PS, ZI, JCP2 at concentrations of 1, 10, 25 and 50 μg/ml for 48 hours. G1, S and G2/M phases are marked with black arrows. Represented were the most prominent samples of 3–5 individual replicates. **B**: Calculation of proliferation and sub-G1 phase. Measurement of proliferation and apoptosis via cell cycle analysis after treatment with the vehicle DMSO (equates to 100%) and the plant extracts JCP1, PS, ZI, JCP2 at concentrations of 1, 10, 25 and 50 μg/ml for 48 h. As proliferative phases the sum of S and G2/M phases were calculated in percentages. As apoptotic fraction the sub G1-peak was measured. (mean ± SD, n = 5, ***P < 0.001, **P < 0.01, *P < 0.5, significantly different compared to control, unpaired *t*-test).

First, the anti-cancer properties of these four extracts were examined by cell cycle analysis. Therefore, we chose the breast cancer cell line MCF-7, a model for hormone-dependent non-invasive cancer types. This model fits for initial screening experiments with these untested extracts. Cell cycle analysis via flow cytometry allowed us to distinguish between alterations in proliferation and apoptosis. Distribution of cell cycle phases (histograms) of MCF-7 cells after treatment with different concentrations (1, 10, 25 and 50 μg/ml) of the plant extract are mentioned in Figure 1A. In untreated, exponentially growing MCF-7 cells the G1- and G2/M-phases were well defined with a large number of dividing cells (S-phase between G1- and G2/M-peaks). Demonstrated were the most prominent histograms of all measurements (Figure 1A).

All four plant extracts caused a significant influence on the proliferation phases of the MCF-7 cells, which turned out to be concentration dependent but not always linear. To quantify proliferation we defined the cell cycle phases S and G2/M as proliferative phases so that the sum of it describes the proliferation rate. In parallel, the apoptotic rates were measured by determination of the sub G1-peak showing DNA fragmentation events. Both results were displayed in Figure 1B. As negative control the DMSO treated cells were set to 100%. As positive controls genistein was used which results were previously reported [17]. Only the PS extract mediated an almost linear reduction in proliferation. In contrast, the ZI extract showed a biphasic effect. Low concentrations (1–10 μg/ml) caused an increase in the proliferative phases and at a concentration >25 μg/ml, the proliferation was significantly reduced. JCP1 induced only a slight proliferation induction after an exposure of 25 μg/ml accompanied by a significant elevation of sub-G1 positive cells. This effect of proliferation and apoptosis induction at the same time is noted by a number of studies have which have shown that cell-cycle regulators could interconnect with proliferation and apoptosis [21]. In contrast, JCP2 displayed a bi-phasic effect on the distribution of the MCF-7 cell cycle phases. At the lowest concentration of 1 μg/ml the content of the G2/M phase nearly doubled in comparison to the DMSO control. Analogously to the PS extract, a JCP2 concentration of 10 μg/ml caused also a significant reduction of G2/M phase and an arrest in G0/G1 phase.

But ultimately, all four extracts mediate a significant increase in sub-G1 phase. The extracts JCP1 and JCP2, already caused at the low concentration of 10 μg/ml a significant increase in the sub-G1 phase. In summary, all four plant extracts were able to influence the proliferation rates of the estrogen receptor-positive cell line MCF-7 depending on the concentrations used. This phenomenon is not uncommon for some effective anti-cancer extracts. For example, soy ingredients like genistein causes biphasic effects on hormone-dependent cancer cell lines [22]. At low concentrations (1–10 μM), genistein stimulates cell proliferation whereas higher concentrations are able to induce a block in G2/M phase. This result suggests that the plant extracts contain substances that trigger similar biphasic effects, known as phytoestrogens such as genistein. In subsequent experiments, the exact ingredients of the extracts by high-performance liquid chromatography (HPLC) and gas chromatography–mass spectrometry (GC-MS) will be analyzed to obtain a deeper insight into the existing drug classes.

**Figure 2:**
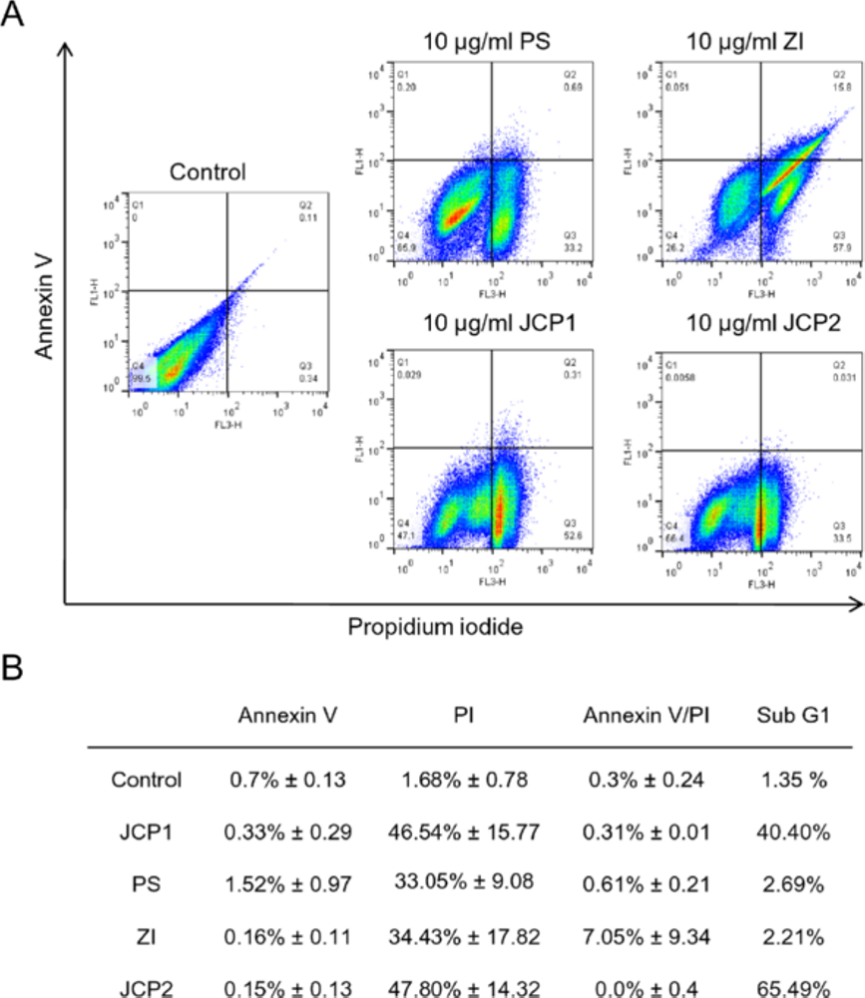
**Apoptosis determination via Annexin V/PI staining. A**: Histogramms of Annexin V/PI- stained MCF-7 cells after treatment with 10 μg/ml of the four plant extracts in comparison with the control treatment for 48 h. Annexin V in conjunction with PI staining was used to distinguish early apoptotic (Annexin V- positive, PI negative; quadrant 1 of each panel) from late apoptotic or necrotic cells (Annexin V positive, PI positive; quadrant 2 of each panel). **B**: Table of quantitative analysis of single Annexin V or PI positive and double positive stained MCF-7 cells. Notably, sub-G1 phase positive cells from the cell cycle measurement were listed for comparison. Results are representative of three separate experiments.

However, to confirm the results of the cell cycle analysis, three additional methods were used to verify the influence on proliferation and apoptosis: Annexin V/PI labeling, TUNEL assay and western blotting experiments of relevant proteins. Beside the calculation of the sub-G1-peak by cell cycle analysis, apoptosis/necrosis induction was determined by using Alexa Fluor488 Annexin V/PI staining (Figure 2). Treatment with 10 μg/ml JCP1 and JCP2 resulted in the highest levels of dead MCF-7 cells (46% and 47%) which were positive for the PI labeling. These values are comparable with the apoptosis data obtained from the determination of the sub-G1 phase in the cell cycle measurement which indeed showed the highest percentages in sub-G1 phase. The treatment with 10 μg/ml PS and ZI resulted in approximately 33% cell death compared to the control. Surprisingly, only the treatment with 10 μg/ml ZI showed a moderate Annexin V/PI labeling, as an indication of late apoptosis induction. PS, and especially JCP1 and JCP2 induced only necrotic events, verified by high amounts of PI-positive cells. These results are similar to those of the TUNEL measurements (Additional file 1: Figure S1). At concentrations of 10 μg/ml PS, JCP1 as well as JCP2 caused DNA fragmentation within the cell nucleus, whereas ZI induced signals within the cytoplasm, indicating for extrinsic death signals.

**Figure 3:**
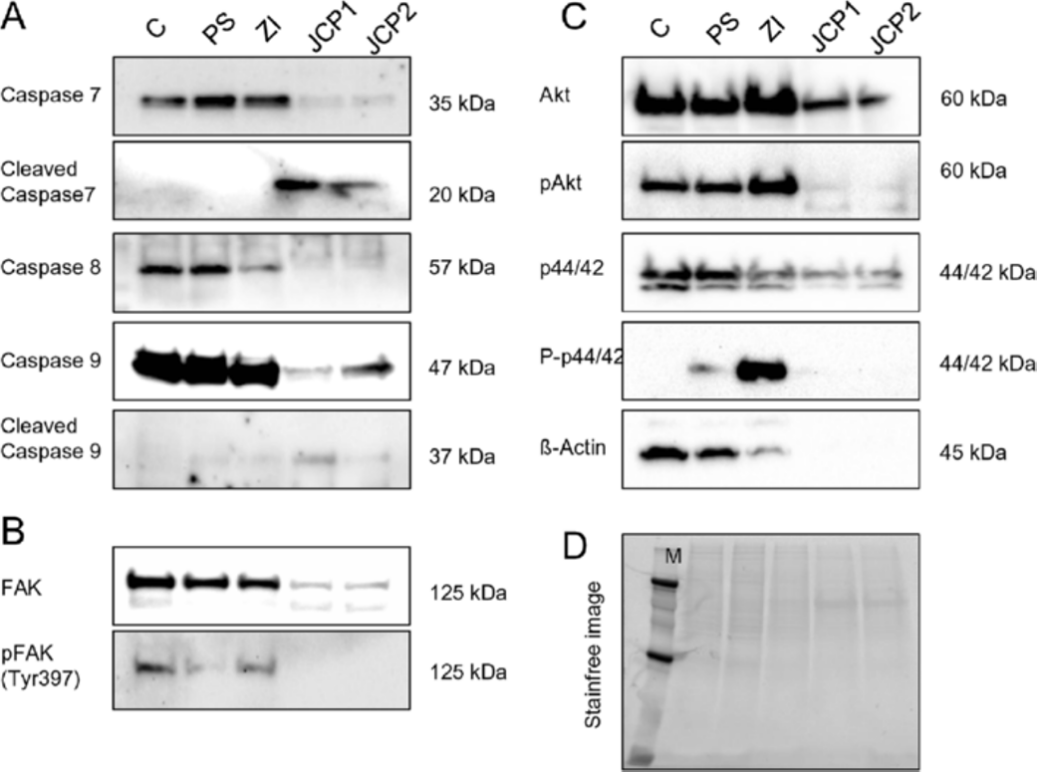
**Protein expression analysis of MCF-7 cells by Western blotting after treatment with 10 μg/ml of the four plant extracts in comparison with the control treatment for 48 h. A**: Determination of caspase 7, 8, and 9 cleavage. Detected were the pro-caspases as well as the cleaved proteins. **B**: Expression of total focal adhesion kinase (FAK) protein and phosphorylated FAK protein at residue Tyr397. **C**: Protein content of kinases: total Akt, phosphorylated Akt at residue S473 (pAkt), total MAPK p44/42, phosphorylated MAPK p44/42 at residues T202/204 (P-p44/42) and β-Actin which did not function as housekeeping protein. **D**: Stain-free image of separated proteins after SDS-PAGE to ensure equal protein amounts on each polyacrylamide gel used.

Furthermore, the induction of apoptosis was verified by western blot analysis with apoptosis relevant specific antibodies (Figure 3A). The treatment with 10 μg/ml JCP1 and JCP2 revealed a cleavage of caspase 7 and caspase 9 so that pro-caspases 7 and 9 were hardly to detect which also applies also to caspase 8. Interestingly, neither the PS nor the ZI extract lead to caspase cleavage. The proliferation results of the cell cycle analysis were validated by the expression analysis of PCNA (Proliferating Cell Nuclear Antigen) (Additional file 2: Figure S2). At a concentration of 10 μg/ml ZI induced an increase of the proliferative phases whereas the JCP1 and JCP2 decreased the expression of PCNA. Ultimately, these results demonstrate that the cell cycle analyses were well performed and comparable with Annexin V/PI labeling as well as with expression analysis of PCNA. The JCP1 and JCP2 extracts mediated the strongest decrease in proliferation with a simultaneous induction of cell death.

### **Plant extracts induced cell detachment mediated by decreased**β**1 integrin expression**

**Figure 4:**
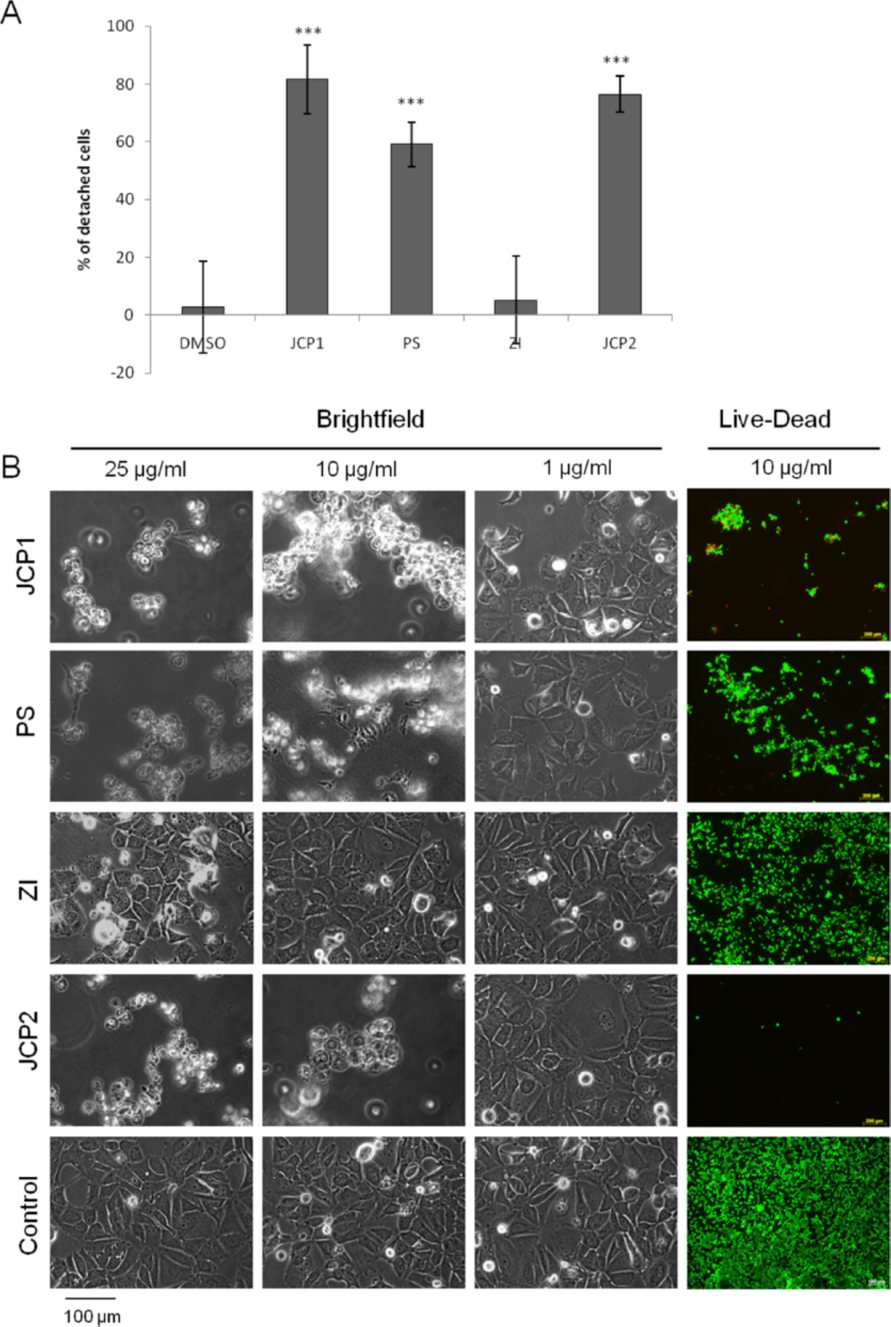
**Measurement of cell detachment and morphological alterations. A**: Cell detachment was calculated by flow cytometry after 48 h after exposure to 10 μg/ml of the plant extracts and 0.1% DMSO. (mean ± SD, n = 8, **P < 0.01, *P < 0.05, significantly different compared to control, unpaired *t*-test). **B**: Bright field microscopy to monitor morphological alterations after treatment with the four plant extracts JCP1, PS, ZI, JCP2 in a concentration dependent manner on MCF-7 cells. Even the lowest concentration of 1 μg/ml caused a significant detachment of the MCF-7 cells from the plate surface. At a concentration of 10 μg/ml almost 70 - 80% of all cells treated with PS, JCP1 and JCP2 were detached. Detachment correlates with induction of apoptosis which was determined by two-color fluorescent Live/Dead-Staining. Green: viable cells; red: dead cells.

Morphological alterations under treatment conditions were verified by bright field microscopy (Figure 4B). The plant extracts PS, JCP1 and JCP2 at concentration of 10 μg/ml or higher induced a loss of cell-cell and cell-matrix adhesion accompanied by rounding of the cells. Treatment with ZI induced no visual cell detachment. Incubation with 1 μg/ml plant extract resulted in a few detached cells while the typical cell cluster of MCF-7, called domes, were not affected. To calculate detachment rates, detached MCF-7 cells after exposure to 10 μg/ml plant extract were counted by flow cytometry (Figure 4A). The extracts JCP1 and JCP2 induced significant increased cell detachment with rates of 81% and 76%, respectively. Exposure with the PS extract resulted in 59% detached cells. The ZI extract caused no significant cell detachment in comparison to the control.

Detachment from the extracellular matrix or lost contact with the neighbor cells is a clear indication of induction of an apoptotic process that is termed anoikis [23]. This observation confirmed the results of the cell cycle measurements that showed a significant increase of apoptotic cells after treatment with 50 μg/ml of the four plant extracts. To confirm the induction of anoikis, which is accompanied by cell detachment, we performed live/dead staining of the cell after exposure to 10 μg/ml of the extracts. These staining demonstrated that only attached cells were viable (green fluorescence) while detachment by the extracts caused cell death (red fluorescence). Moreover, this test showed that the concentration of 10 μg/ml of each extract led to a different degree of cell detachment. Treatment with JCP1 and JCP2 resulted in the highest cell detachment rates (approximately 60 - 90%), so that nearly no viable cells were attached to the cell culture plates. The ZI extract exhibited the lowest rate of cell detachment and only a few dead cells, which correlated with the results of the cell cycle measurements where the ZI extract at a concentration of 10 μg/ml showed a significantly increased proliferative phase.

**Figure 5:**
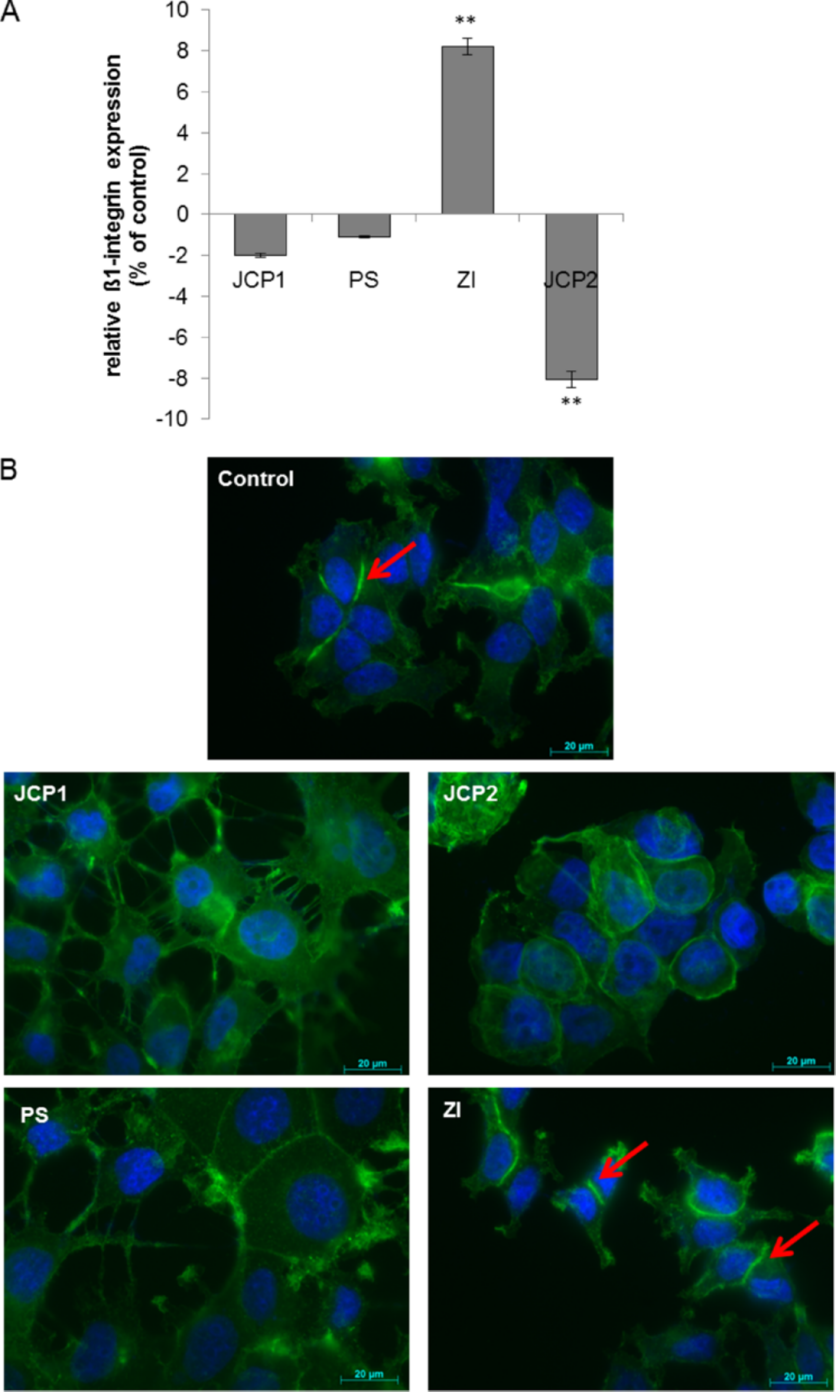
**Monitoring of adhesion related proteins. A**: Cell membrane associated (cell surface) β1-integrin expressions of plant extract treated (10 μg/ml) MCF-7 cells in percent of control measured by flow cytometry. As control DMSO treated cells were used. (mean ± SD, n = 3, **P < 0.01, *P < 0.05, significantly different compared to control, unpaired *t*-test). **B**: Fluorescence microscopy of F-actin (green) alterations after treatment with 10 μg/ml of the plant extract. Nuclei are marked in blue. Arrows point to the increased actin accumulation at sides of cell-cell contacts.

The ability of epithelial cells to survive through suppression of anoikis depends on their engagement to the extracellular matrix through a family of heterodimeric transmembrane receptors named integrins. Anoikis in mammary epithelial cell can be initiated by direct inhibition of β1-integrins [24, 25]. Therefore, the β1 integrin expression by flow cytometry analysis was measured (Figure 5A). Although the three plant extracts, JCP1, JCP2 and PS lead to cell detachment at a concentration of 10 μg/ml, only JCP2 lowered the β1-integrin expression in MCF-7 cells, significantly. PS and JCP1 revealed only a slight decrease in β1-integrin expression while ZI increased the β1-integrin level significantly up to 9%. Because the flow cytometry data only represent the levels of membrane associated β1-integrin expression, western blotting was performed with a β1-integin specific antibody. Additional file 2: Figure S2 shows the expression pattern of β1-integrin in the soluble and membrane protein fractions. Consistent with flow cytometry results the exposure with 10 μg/ml ZI revealed a significant increase of β1-integrin in the membrane fraction, while the soluble content was markedly reduced. The extracts JCP1 and JCP2 convey no significant change in β1-integrin expression, neither in the soluble nor the membrane fraction. The PS extract is the exception. Compared with the flow cytometry data 10 μg/ml PS extract caused a significant increase in β1-integrin expression in the membrane fraction while the soluble protein content was reduced. This phenomenon may be attributable to the fact that different antibodies were used for the flow cytometry analysis. The antibody suitable for western blotting detection of β1-integrin was raised against amino acids 375–480 mapping within an extracellular domain of β1-integrin of human origin (sc-374429, Santa Cruz). The flow cytometric antibody (CD29, Immunotech) was raised against the entire amino acid sequence, making it more specific for β1-integrin detection. Thus, it is possible that there will be slight differences in the recognition of the β1-integrin protein. Accordingly, the western blot results are only a general review of the flow cytometry data. However, it could be confirmed that the ZI extract mediates a clear overexpression of β1-integrin in the cell membrane. In contrast, JCP1 and JCP2 caused a slight reduction in β1-integrin expression.Additionally, the expression levels of the focal adhesion kinase (FAK), a widely expressed cytoplasmic protein tyrosine kinase involved in integrin-mediated signal transduction and its phosphorylation status was checked (Figure 3B). Consistent with the lowered β1-integrin expression levels after treatment with JCP1 and JCP2, the expression of total FAK and the phosphorylation at residue Tyr397 decreased. Also the exposure to PS caused a slight decrease in the autophosphorylation of FAK indicating for the deactivation of FAK and lowered adhesion to the extracellular matrix.

These findings suggest that the plant extracts target different signaling pathways in the MCF-7 cells and thus initiate various mechanisms of cell detachment. Loss of integrin binding to extracellular matrix proteins results in impairment of cell spreading which depends on the integrity of the internal actin cytoskeleton formation [26]. This assertion is supported by the fact that the formation of the actin cytoskeleton is affected differently by the plant extracts (Figure 5B). Under control conditions MCF-7 cells form a diffuse actin cytoskeleton without any visible stress fibers. The actin filaments are short and find to be enriched at sides of cell-cell contacts (Figure 5B; red arrows). JCP1, JCP2 and PS revealed a distinct alteration of the actin organization. No strong accumulation of actin in adjacent cells could be observed in comparison with controls. By treatment with the PS and JCP1 strong cellular contacts were diminished. Cells seem to be enlarged in their areas and express filopodia. Consistent with the increased β1-integrin expression by ZI increased formation of actin filaments was observed between neighboring cells (Figure 5B, red arrows). In addition, the western blot results confirm that after treatment with JCP1 and JCP2 the β-actin filaments were degraded: further evidence for the induction of anoikis (Figure 3C).However, the three plant extracts (JCP1, JCP2 and PS) harbor anticancer potential, which should be analyzed in further studies. Especially the signaling mechanisms should be identified because initial analysis of central signaling molecules like the serine/threonine-specific protein kinase Akt and mitogen-activated protein kinases (MAPKs) showed alterations in their expression levels (Figure 3C).

### Plant extracts harbor IC_50_ values between 23 and 38 μg/ml

**Figure 6:**
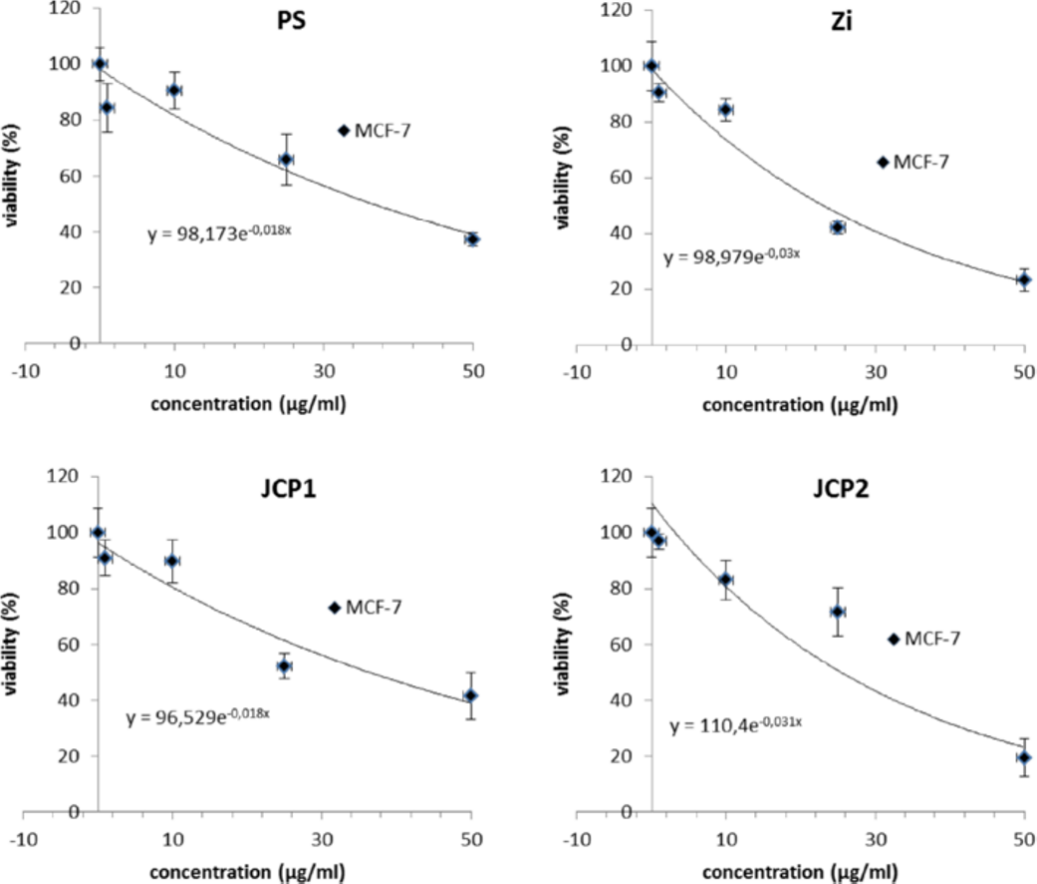
**Determination of IC**_**50**_ **values.** The 50% inhibition concentration (IC_50_) values of the four plant extracts JCP1, PS, ZI, JCP2 on MCF-7 cancer were measured by MTS assay. The four extracts reduced viability of MCF-7 cells in a dose dependent manner. IC_50_ values (see Table 1) were calculated from the dose response curves by nonlinear regression. Background absorbance was corrected against MTS reaction without cells and control treatment (0.1% DMSO).

Finally, the IC_50_ values of the plant extracts for MCF-7 via MTS assay was determined (Figure 6). Data of the MTS measurements were plotted on dose response curve and IC_50_ values were calculated by non-linear regression. The IC_50_ values for JCP1, PS, ZI and JCP2 for MCF-7 cells were 36.55, 37.36, 22.76 and 25.55 μg/ml, respectively. These values describe a moderate potential as an anti-cancer agent but colorimetric test were not that convincing and require confirmation by additional experiments [27]. Also, because the cell cycle analysis, bright field microscopy and β1-integrin expression revealed a markedly lower effective concentrations (1–10 μg/ml) of the plant extracts. However, further investigation is needed to explore the detailed anticancer potential of these plant extracts and the corresponding signaling pathways.

## Conclusion

Anticancer screening experiments for bioactive agents from natural products are in focus of current research. But effective screening methods are rare and sometimes difficult to interpret. Therefore in this study standard methods were chosen for the interpretation of the influence on proliferation (cell cycle measurement, PCNA expression by Western blotting), apoptosis (cell cycle measurement, Annexin V/PI labeling, TUNEL assay, live-dead staining, caspase claevage), morphology (bright field imaging), metabolism (MTS assay) and adhesion (β1-integrin expression, F-actin staining, cell detachment). These methods allow an overview of the potential effectiveness of the extracts on the key mechanisms of cancer cells, but do not allow a clear identification of signaling pathways. All four plant extracts displayed distinct alterations on these major cellular mechanisms in the breast cancer cell line MCF-7. These results reveal an anti-tumorigenic potential of the four plant extract. But the dose response curves and calculated IC_50_ values also indicate a general cytotoxic activity. This could be due to the mixture of ingredients within the extracts. Accordingly, in subsequent experiments the active substance classes will be identified and fractionated. The overall goal for future work is to isolate the active compounds and their testing on different cancer cell lines.

Interestingly, the PS, JCP1 and JCP2 plant extracts induced cell rounding and cell detachment at concentrations ≥10 μg/ml. Cell detachment could be caused by several processes. One protein relevant for cancer cell progression and metastasis is the β1-integrin [28]. This finding implicates that the ZI extracts influences different cellular pathway compared with other extracts. However, the four tested plant extracts exert cytotoxic and anticancer potential which should be investigated in further studies.

In conclusion, the results in the present study coincide to some extent with the traditional uses of the plants investigated. Our results further supported the idea that medicinal plants can be promising sources of potential anticancer, antimicrobial and antioxidants agents. The present results will form the basis for selection of plant species for further investigation in the potential discovery of new natural bioactive compounds. Studies aimed at the isolation and structure elucidation of anticancer chemical constituents are in progress.

## Electronic supplementary material

Additional file 1: Figure S1: DNA fragmentation by TUNEL assay. Late apoptotic effects induced by 1 and 10 μg of the four plant extracts on MCF-7 cells were analyzed by TUNEL assay to measure the extent of DNA fragmentation visualized by confocal laser scanning microscopy (LSM 780, Carl Zeiss, Jena, Germany). Green fluorescence within the cell nucleus of PS, JCP1 and JCP2 (10 μg/ml) reflect DNA damage. Notably, ZI extract causes cytosolic labeling, an indication for extrinsic apoptotic pathways. (TIFF 1 MB)

Additional file 2: Figure S2: PCNA and integrin expression by western blotting. Expression analysis of β1 integrin and Proliferating Cell Nuclear Antigen (PCNA) of the soluble and membrane fraction of MCF-7 cells after treatment with 10 μg/ml plant extract in comparison with the DMSO control. Loading controls were visualized by stain-free imaging of the SDS-PAGEs prior blotting procedure. Note that β1 integrin expression is demonstrated twice. Upper panel shows the normal exposure, lower panel the overexposed variant. (TIFF 549 KB)
