# Supplementary figures and images for: Pro-apoptotic and anti-adhesive effects of four African plant extracts on the breast cancer cell line MCF-7
Source: BMC Complement Altern Med. 2014 Sep 9;14:334. doi: 10.1186/1472-6882-14-334 (PMC4177160; doi:10.1186/1472-6882-14-334)

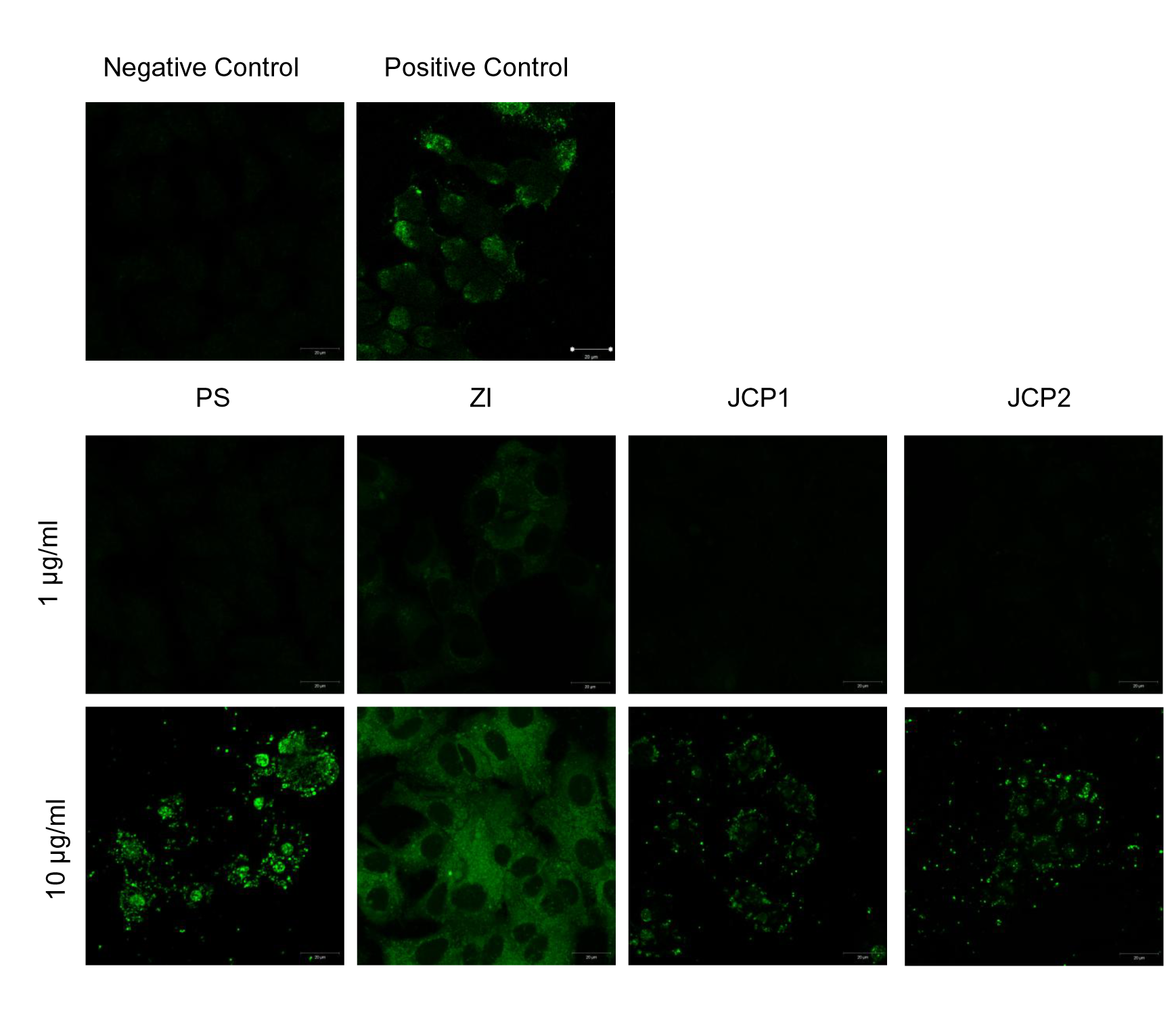

Supplement: Supplementary file 1 — Additional file 1: Figure S1: DNA fragmentation by TUNEL assay. Late apoptotic effects induced by 1 and 10 μg of the four plant extracts on MCF-7 cells were analyzed by TUNEL assay to measure the extent of DNA fragmentation visualized by confocal laser scanning microscopy (LSM 780, Carl Zeiss, Jena, Germany). Green fluorescence within the cell nucleus of PS, JCP1 and JCP2 (10 μg/ml) reflect DNA damage. Notably, ZI extract causes cytosolic labeling, an indication for extrinsic apoptotic pathways. (TIFF 1 MB) [file 12906_2013_1912_MOESM1_ESM.tiff]

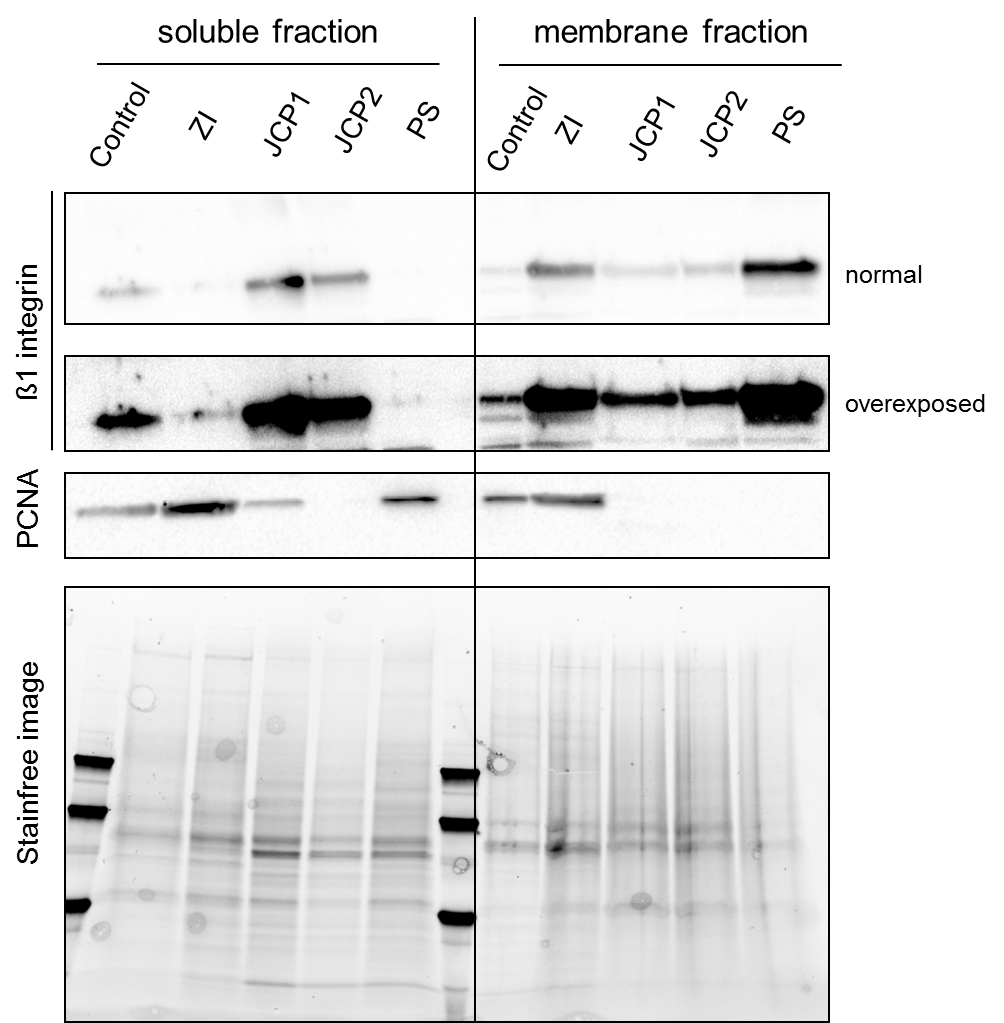

Supplement: Supplementary file 2 — Additional file 2: Figure S2: PCNA and integrin expression by western blotting. Expression analysis of β1 integrin and Proliferating Cell Nuclear Antigen (PCNA) of the soluble and membrane fraction of MCF-7 cells after treatment with 10 μg/ml plant extract in comparison with the DMSO control. Loading controls were visualized by stain-free imaging of the SDS-PAGEs prior blotting procedure. Note that β1 integrin expression is demonstrated twice. Upper panel shows the normal exposure, lower panel the overexposed variant. (TIFF 549 KB) [file 12906_2013_1912_MOESM2_ESM.tiff]
